# The effect of consequent exposure of stress and dermal application of low doses of chlorpyrifos on the expression of glial fibrillary acidic protein in the hippocampus of adult mice

**DOI:** 10.1186/1745-6673-6-4

**Published:** 2011-03-08

**Authors:** Kian Loong Lim, Annie Tay, Vishna Devi Nadarajah, Nilesh Kumar Mitra

**Affiliations:** 1 Postgraduate & Research Department, International Medical University, No.126, Jalan 19/155B, Bukit Jalil, 57000, Kuala Lumpur, Malaysia; 2 Pathology Department, International Medical University, No.126, Jalan 19/155B, Bukit Jalil, 57000, Kuala Lumpur, Malaysia; 3 Human Biology Department, International Medical University, No.126, Jalan 19/155B, Bukit Jalil, 57000,Kuala Lumpur, Malaysia

## Abstract

**Background:**

Chlorpyrifos (CPF), a commonly used pesticide worldwide, has been reported to produce neurobehavioural changes. Dermal exposure to CPF is common in industries and agriculture. This study estimates changes in glial fibrillary acidic protein (GFAP) expression in hippocampal regions and correlates with histomorphometry of neurons and serum cholinesterase levels following dermal exposure to low doses of CPF with or without swim stress.

**Methods:**

Male albino mice were separated into control, stress control and four treatment groups (n = 6). CPF was applied dermally over the tails under occlusive bandage (6 hours/day) at doses of 1/10th (CPF 0.1) and 1/5th dermal LD_50 _(CPF 0.2) for seven days. Consequent treatment of swim stress followed by CPF was also applied. Serum cholinesterase levels were estimated using spectroflurometric methods. Paraffin sections of the left hippocampal regions were stained with 0.2% thionin followed by the counting of neuronal density. Right hippocampal sections were treated with Dako Envision GFAP antibodies.

**Results:**

CPF application in 1/10th LD_50 _did not produce significant changes in serum cholinesterase levels and neuronal density, but increased GFAP expression significantly (p < 0.001). Swim stress with CPF 0.1 group did not show increase in astrocytic density compared to CPF 0.1 alone but decreased neuronal density.

**Conclusions:**

Findings suggest GFAP expression is upregulated with dermal exposure to low dose of CPF. Stress combined with sub-toxic dermal CPF exposure can produce neurotoxicity.

## Background

Almost 85% of the 2.6 million metric tonnes of active components of pesticides manufactured every year is used in commercial farming [1]. Occupational pesticide poisoning is an important risk factor for farmers as they are constantly being exposed to pesticides. It has also been found that most occupational exposures are dermal [2]. CPF (O, O-diethyl O-3, 5, 6-trichloro-2-pyridyl phosphorothioate) is a broad spectrum organophosphate pesticide. It inhibits the enzyme cholinesterase by binding irreversibly to, and phosphorylating its active site. A report in 2001 by the United States Environmental Protection Agency on pesticide use approximates that 50 to 60% of the total 11-16 million pounds of CPF used in the US was for agriculture [3].

In chronic low-level exposures of CPF, crop workers recorded a reduced performance in neurobehavioral tests [4]. Individuals with histories of exposure to low, sub-clinical levels of chlorpyrifos have also reported reduced levels of concentration, word finding and short-term-memory impairment [5]. CPF has also been reported to produce neurobehavioral and morphological damages in the nervous systems of animals during embryonic life through to postnatal development [6,7]. Previous work by the authors has found that sub-toxic doses (1/5th and 1/2 LD_50_) of chlorpyrifos applied dermally for 3 weeks can produce significant hippocampal neuronal loss, and that stress can exacerbate this damage [8]. Even the inhibition of serum cholinesterase which was reduced by 76% with dermal application of CPF in the dose of 1/5th dermal LD_50 _for 21 days got exaggerated by addition of swim stress at 38°C by 19.7%.

The biological efficacy of many toxicants can be exacerbated by exposure to heat stress [9]. Administration of pyridostigmine, a carbamate AChE inhibitor normally impermeable to the blood brain barrier (BBB), during the Persian Gulf War, resulted in an increase in the occurrences of reported CNS symptoms by more than threefold, indicating a possible link between stress and increased BBB permeability [10].

One of the proteins associated with neuronal damage is glial fibrillary acidic protein (GFAP). GFAP is a cytoplasmic intermediate filament protein found in astrocytes. They maintain the structural integrity of astrocytes, especially when these cells undergo hypertrophy and hyperplasia in response to a non-invasive CNS injury [11] whereby, expression of GFAP is upregulated [12]. A characteristic feature of gliosis, GFAP upregulation often occurs in response to injury in the brain [13]. Numerous neurological studies have associated CNS damage with increased GFAP expression [12,13]. It has also been suggested that GFAP is a sensitive and early biomarker of neurotoxicity, its up-regulation preceding anatomically perceptible damages in the brain [14-16]. Predominantly, only studies investigating developmental or in utero exposure to CPF have estimated GFAP expression [17,18]. The effect of dermal application of CPF on GFAP expression in the hippocampus has not been reported.

The aim of this study was to determine the expression of GFAP in the hippocampal region of adult mice following consequent exposure of repeated stress and dermal application of low dose chlorpyrifos for small duration (7 days), and to correlate the findings with changes in serum cholinesterase and neuronal density of Cornu Ammonis of hippocampus. The study aimed to look into the changes in the above parameters with reference to our previous findings [8] with dermal application of subtoxic doses of CPF with swim stress for a relatively prolonged period of 21 days.

## Methods

Commercial preparations of CPF (O, O-diethyl O-3, 5, 6-trichloro-2-pyridyl phosphorothioate), Zespest, manufactured in Kuala Lumpur, Malaysia was used in this study. This preparation contained 38.7% W/W CPF diluted in xylene. The mixture was further diluted in xylene to prepare doses of 1/10th LD_50 _(20.2 mg/kg body weight CPF in 1 mL) and 1/5th LD_50 _(40.4 mg/kg body weight CPF in 1 mL) CPF solution.

Male Swiss albino mice (species: ICR), 60 days old (30-32 g) were used in this study. They were housed in plastic cages (six in a cage) and were exposed to natural, twelve-hourly light and dark sequence. Lab chow (pellet feed) and water were given *ad libitum*. Animal experiments adhered to the principles stated in the guide-book of laboratory animal care and user committee of the International Medical University and in accordance with the declaration of Helsinki. The mice were divided into six groups (n = 6). Control group was applied with xylene, CPF0.1 group was applied with 1/10th LD_50 _of CPF and CPF0.2 group was applied with 1/5th LD_50 _of CPF. Swim stress at 38°C followed by application over the tails with xylene (Control s), 1/10th LD_50 _CPF (CPF 0.1 s) and 1/5th LD_50 _CPF (CPF 0.2 s) was also done. All the 6 groups were used in the experiment which lasted 1 week only.

CPF solution was applied directly to the tail of the mice under occlusive bandage. Animals were exposed to CPF daily for 1 week. Absorptive surgical gauze soaked with either xylene (control) or 1 ml of CPF solution (1/10th or 1/5th LD_50_) was wrapped around the tail. Aluminium foil was then wrapped over to prevent vaporisation of the CPF solution. The foil wrappings were left on the tail for 6 hours. After removal of the wrappings, traces of CPF solution were removed by dipping the tails of all mice in clean water.

A plastic container measuring 30 cm × 30 cm × 40 cm was filled with water to a depth of 30 cm. The water was heated to a temperature of 38°C. The animals were then placed in the water for a swim session lasting 6 minutes [19]. After the session of forced-swimming, the mice were dried and allowed to rest for approximately 15 minutes before the CPF solution was applied to their tails as previously described.

Body weight was measured at the beginning and end of both experimental periods. At the end of 7 days, the animals were anaesthetized with intraperitoneal administrations of pentobarbitone. Blood samples were collected for cholinesterase and corticosterone assay. Brain tissues were collected for histomorphometric studies and GFAP immunohistochemical staining.

### Serum cholinesterase assay

The Amplex Red Acetylcholine/Acetylcholinesterase assay kit from Molecular probes Inc. USA (Invitrogen detection technologies, A12217) was used to estimate serum cholinesterase activity using a fluorescence microplate reader. A working solution of 400 μM Amplex Red reagent containing 2 U/mL Horse Radish Peroxidase (HRP), 0.2 U/mL choline oxidase and 100 μM Acetylcholine (ACh) was prepared from the stock solutions. The reaction began when 100 μL of the working solution was added to each well containing the serum samples and controls diluted to 40×. Serum samples and controls were tested in duplicates. Fluorescence emitted by the individual samples was measured in a microplate reader at an excitation of 560 nm and emission detection at 590 nm. Background fluorescence was eliminated by subtracting values derived from the negative control. To obtain a standard curve, cholinesterase concentrations of the standards and their corresponding fluorescence readings were converted to log_10 _values before being plotted against each other. This was done to facilitate regression analysis of the data. Using the standard curve, serum concentrations of cholinesterase from the samples of different groups were then calculated.

### Serum corticosterone assay

The corticosterone enzyme immunoassay (EIA) kit from Cayman Chemicals (No. 10005590) was used. This assay used a corticosterone tracer which was a corticosterone-cholinesterase conjugate. The well-plates were coated with mouse monoclonal anti-rabbit IgG. Corticosterone in the serum sample and the corticosterone tracer provided in the kit compete for limited numbers of corticosterone-specific rabbit anti-serum binding sites. The plates were washed to remove the unbound reagents and then Ellman's reagent was employed to estimate cholinesterase. The colour produced by this enzymatic reaction measured in a fluorescence microplate reader at an absorbance of 405 nm was proportional to the amount of corticosterone-tracer bound to the well. The amount of free corticosterone present in the well was inversely proportional to the amount of emission. The purification of serum samples was not employed as two dilutions, 20× and 40×, showed good correlation in the amount of final corticosterone. The logit (B/B_0_) values were calculated by dividing absorbance values of every standard well (B) by the average value of maximum binding wells (B_0_). To obtain standard curve, concentration standards were plotted against logit values. Logit of the data was then employed in Microsoft Excel to get the serum concentrations of corticosterone by substitution in the linear regression analysis.

### Histomorphometric studies and estimation of GFAP expression

Perfusion of the brains was carried out using 10% formal saline. The area between the optic chiasma and infundibulum in which the hippocampus is located was further dissected followed by paraffin processing. Right sagittal half was used for GFAP immunohistochemical staining. Coronal serial sections from the left half of the hippocampal area, 8 micron thick, were stained with Nissl stain (0.2% thionin in acetate buffer). Every 10^th ^section in each animal was selected. Using Nikon's Brightfield Compound Microscope, YS100 (attached with Nikon camera), the slides were examined and photographed under 400× objective. For each slide, two random areas of CA1, one random area of CA2 and two random areas of CA3 were examined. Neuronal counts were then performed in the regions of the hippocampus as mentioned above within a measured square area of 160 × 160 μm. Only neurons with a clearly defined border and visible single nucleus were counted. 10 random neuronal nuclear diameters were also taken for each region. The neuronal counts were then used to obtain the absolute density (P), of neuronal nucleus per unit area of section using the Abercrombie formula: P = A. M/L+M; M = Section thickness in micron; L = Mean nuclear diameter of respective area; A = Neuronal count [20]. The neuronal density per unit area (mm^2^) was then calculated.

Three stained slides containing hippocampal areas (every 27^th ^section) were chosen for each animal. DakoCytomation Envision+ system together with polyclonal rabbit Anti-glial Fibrillary Acid Protein (GFAP) antibody were used to estimate GFAP expression in the hippocampal sections. This antibody could be used to identify astrocytes by light microscopy. Following dewaxing by xylene, 4 micron thick sections were gradually rehydrated. Then washing was done by Tris-buffered saline with Tween (TBST). The peroxidise was blocked followed by application of anti-GFAP antibody. The polymer was added to bind with the primary antibody which was followed by application of chromogen. Ultimately Haematoxylin counterstain was employed followed by dehydration, clearing and mounting. Using Nikon's Brightfield Compound Microscope, YS100 (attached with Nikon camera), the slides were viewed and photographed under 400× objective. For each slide, three random areas in the stratum moleculare-lacunosum and two random areas in stratum oriens of the hippocampus were examined. The areas of the captured images were constant. Astrocytic cell counts were then performed in the regions of the hippocampus as mentioned above within a measured square area of 200 × 200 μm. Only cells with a clearly defined nuclear border and radiating processes containing GFAP staining were counted. The density of astrocytes per unit area (mm^2^) was then calculated.

### Statistical analysis

Mean serum cholinesterase levels of individual mouse under different groups were subjected to one way ANOVA statistical analysis using SPSS 11.5. Inter-group significance was tested by Post Hoc LSD test, provided ANOVA showed significant difference between the groups. The mean absolute counts (per mm^2^) of the neuronal count and astrocytes were subjected to One Way ANOVA statistical analysis to identify any statistically significant differences in the counts between the treatment groups. Post hoc Bonferroni was employed to determine the level of significance in inter-group difference.

## Results

### Changes in serum cholinesterase

**Figure 1:**
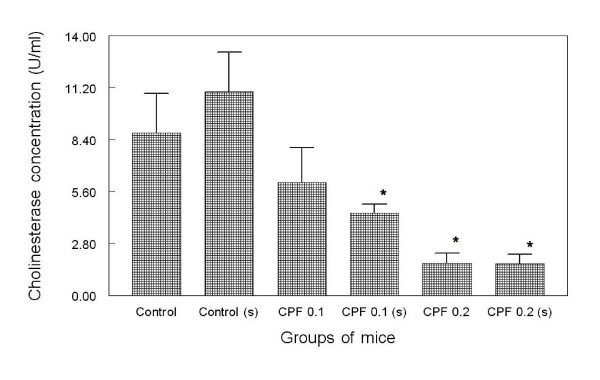
**Bar chart showing mean serum cholinesterase (± SD) concentration in mice groups at the end of experiment**. The results are derived from 40× dilution of the samples. One way ANOVA shows F (5, 29) = 9.73, p < 0.05 * indicates significant difference (p < 0.05) compared to the control group in post hoc LSD test. Error bars indicate ± standard deviations.

Cholinesterase activity was reduced by 30.5% with exposure to CPF in 1/10th dermal LD_50 _compared to the normal group. With dermal application of CPF in 1/5th LD_50 _for 7 days, a significant reduction (p < 0.05, One way ANOVA, post hoc LSD) by 80.25% in serum cholinesterase activity was observed (Figure 1). Thus a dose-dependent depletion in the activity of serum cholinesterase was observed. Swim stress followed by dermal CPF application caused further depletion in cholinesterase activity by 19.3% (CPF 0.1 s) and 0.4% (CPF 0.2 s) respectively compared to CPF 0.1 (1/10th LD_50_) and CPF 0.2 (1/5th LD_50_) groups. However, both these changes observed in swim stress groups were not statistically significant. Application of swim stress for 7 days in control (s) group, increased serum cholinesterase activity by 25% compared to the control group. Both the groups with stress + application of CPF (CPF 0.1 s and CPF 0.2 s) showed statistically significant reduction in cholinesterase activity (p < 0.05, One way ANOVA, post hoc LSD) compared to the control and swim stress only group (Control s).

### Changes in serum corticosterone

**Figure 2:**
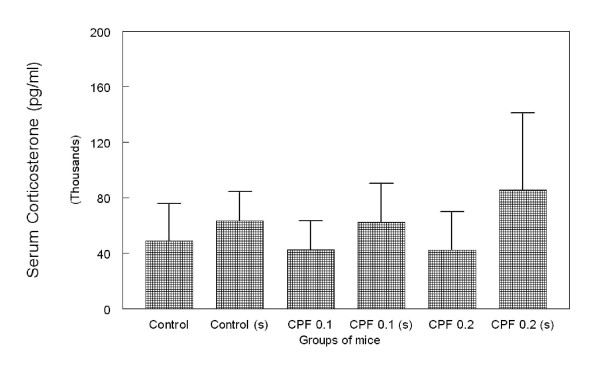
**Bar chart showing mean serum corticosterone (± SD) concentration in mice group at the end of experiment**. The results are derived from 40× dilution of the samples. Error bars indicate ± standard deviations.

Elevation of serum corticosterone levels confirmed that forced swim stress daily for six minutes was sufficient to induce stress in the experimental mice. While application of both doses of CPF failed to increase serum corticosterone, the groups with swim stress (control s, CPF 0.1 s, CPF 0.2 s) showed higher corticosterone levels compared to the control group (Figure 2) by 30%, 27.8% and 43% respectively. Mice group with only swim stress showed 30% increase in the serum corticosterone level compared to the control.

### Changes in histological and histomorphometric studies

Upon qualitative observations of the hippocampal pyramidal neurons, the group receiving 1/10th LD_50 _CPF for 1 week (CPF 0.1) showed only few pyknosed neurons. Quantitative study showed that the neuronal count reduced significantly (p < 0.05) compared to the control group only in CA3 hippocampal region (Table 1). In contrast, when swim stress was applied prior to CPF exposure at the same dose (CPF 0.1 s), substantially more pyknosed neurons were observed in CA1 and CA3 areas of the hippocampus and the neuronal count reduced significantly (p < 0.05) compared to the control group (Table 1). At the higher dose of 1/5th LD_50 _CPF with or without stress (CPF 0.2 and CPF 0.2 s), pyknosis of pyramidal neurons as well as areas of vacuolation were observed in all three areas of hippocampus. The changes observed with swim stress (CPF 0.2 s) were significant compared to the control but was not significant compared to CPF 0.2. Quantitative observations of the hippocampal pyramidal neurons showed that application of 1/10th LD_50 _CPF for 1 week (CPF 0.1) failed to significantly reduce neuronal density in the CA1 and CA2 areas of the hippocampus (7.60% and 13.61% reduction respectively). In CPF 0.1 s group where swim stress was applied in conjunction with CPF, a significant reduction in neuronal density was now observed (15.11% and 20.55% reduction respectively) compared to the control. However the reduction observed in neuronal count was not significantly different from CPF exposure alone (CPF 0.1).

**Table 1:** Table showing mean (± SD) neuronal density in mice groups in different hippocampal areas at the end of the experiment

Experimental Group	Absolute neuronal density in CA1 ( per mm^2^)	Absolute neuronal density in CA2 ( per mm^2^)	Absolute neuronal density in CA3 ( per mm^2^)
**Control**	881.8 ± 134	710.5 ± 146	640.7 ± 75
**Control(s)**	902.1 ± 124	662.9 ± 156	640.6 ± 95
**CPF 0.1**	814.7 ± 158	613.8 ± 125	504.3* ± 116
**CPF 0.1(s)**	748.5* ± 185	564.5 ± 100	501* ± 119
**CPF 0.2**	768.7* ± 201	578.7* ± 103	483.3* ± 167
**CPF 0.2(s)**	746.6* ± 163	525.7* ± 96	467.7* ± 119

Footnote:

* indicates significant difference (p < 0.05) compared to the control group in One Way ANOVA followed by Post Hoc Bonferroni test.

### Changes observed in GFAP immunostaining

**Figure 3:**
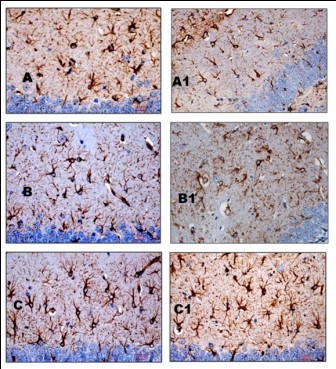
**Photomicrograph showing the immunohistochemical staining of GFAP expression in stratum moleculare-lacunosum of the hippocampus in groups of mice at the end of experiment**. Brown colour rounded cells with processes are the astrocytes. A-Control group; A1-Control (s) group; B-CPF0.1 group; B1-CPF 0.1 (s) group; C-CPF 0.2 group; C1-CPF 0.2 (s) group. (GFAP, 400×)

Examination of the photomicrographs revealed that following one week of application, longer and more numerous astrocytic processes were observed in CPF 0.2 group compared to CPF 0.1 (Figure 3C and 3B respectively) in stratum moleculare and stratum Oriens. Quantitative study showed that the astrocytic density was raised in all groups receiving CPF applications (Table 2). An increase of 37.21% in astrocytic density was observed in CPF 0.1 group (Figure 3B) compared to the control (Figure 3A), while a further increase was seen in CPF 0.2 (41.08%) group (Figure 3C). When stress and CPF at doses of 1/10th dermal LD_50 _were applied concurrently (Figure 3B1), astrocytic density was not increased compared to CPF at 1/10th dermal LD_50 _alone (Figure 3B). Increase in astrocytic density in CPF 0.2 s (47.40%) (Figure 3C) was greater compared to CPF 0.2 (41.08%) (Figure 3C1). One way ANOVA followed by Post Hoc tests (Bonferroni) showed that groups CPF 0.1, CPF 0.1 s, CPF 0.2 and CPF 0.2 s differed significantly from the control group (p < 0.001) in the counts of astrocytic density in Stratum Moleculare-lacunosum of hippocampus but in Stratum Oriens, only CPF 0.1, CPF 0.2 and CPF 0.2 s differed significantly from the control group (p < 0.05). In both the areas, no statistically significant increase in astrocytic density was found in CPF 0.1 s and CPF 0.2 s groups compared to CPF 0.1 and CPF 0.2 groups.

**Table 2:** Table showing the mean (± SD) astrocytic density in stratum moleculare-lacunosum and stratum oriens of hippocampus in mice groups at the end of experiment

Groups	Stratum Moleculare-lacunosum ( per mm^2^)	Stratum Oriens ( per mm^2^)
**Control**	256.9 ± 53	125 ± 54
**Control (s)**	284.3 ± 67	125 ± 54
**CPF 0.1**	352.6 ± 99*	193.3 ± 62*
**CPF 0.1(s)**	343.9 ± 85*	164.7 ± 64
**CPF 0.2**	362.5 ± 96*	202.8 ± 91*
**CPF 0.2(s)**	378.7 ± 83*	241.2 ± 64*

Footnote:

* indicates significant difference (p < 0.001) compared to the control in One Way ANOVA followed Post Hoc Bonferroni test.

## Discussion

Following 1 week of application of CPF, mean body weights of the mice receiving dermal applications of CPF were reduced compared to the control group. The weight loss observed in this study could be attributed to the effect of CPF causing cholinergic overstimulation, leading to increased gastric motility and a reduction in absorption [21]. Furthermore, cholinergic overstimulation of nicotinic receptors can cause increased muscular activity (fasciculation and tremors) and thus increases energy consumption. Daily applications of CPF for seven days at the lower dose (1/10th dermal LD_50_) could reduce plasma cholinesterase activity compared to control group (without stress, 30.5% reduction and with stress, 49.8% reduction) but the reduction in CPF only group was not statistically significant. The reduction in stressed group was statistically significant compared to the control. Similar to the findings in this study, a previous study found that subsequent to daily low dose (12% LD_50_) injection of the OP soman to mice for three days, plasma cholinesterase activities were inhibited by 32% [22]. In a separate study, 14 days after dermal applications of OP diisopropylfluorophosphate (DFP) to monkeys at low doses of 0.01 mg/kg BW, cholinesterase activity was reduced by 76% [23]. A single dermal application of CPF in humans for four hours absorbed CPF very little (4.3%), and CPF was not completely eliminated from the body even after 120 hours, suggesting accumulation of CPF in the body [24]. The CPF applied in this study was dissolved in the xylene, which is an organic solvent. As organic solvents dissolve fat, xylene can be easily absorbed by the layer of fat in the skin.

Compared to the previous study by this author [8], where application of swim stress and CPF (1/5^th ^LD_50_) for 21 days facilitated the reduction in serum cholinesterase by 19.7% (compared to only application of CPF), this study showed that application of stress for lower duration (7 days) with lower dose of CPF (1/10th LD_50_) can bring down the serum cholinesterase levels by similar level (19.3%). The shorter duration of stress might not have potentiated the neurotoxic effects of CPF enough in all the mice. Hence the changes observed with stress remained statistically insignificant compared to the CPF only groups but became significant compared to the control.

Qualitative observations of the hippocampal neurons in this study showed that following seven days of low dose CPF application (1/10th dermal LD_50_), no apparent damage to the neurons was visible. However at the higher dose (1/5th dermal LD_50_), seven days of application resulted in visible damage in the form of pyknosis. Dendritic morphology was assessed in the prefrontal cortex, CA1 area of the hippocampus and the nucleus accumbens following repeated (14 days) low dose intraperitoneal application of OP malathion (40 mg/kg BW) in mice. Dendritic length in the hippocampus and prefrontal cortex, and density of dendritic spines in all the three areas assessed were reduced [25]. As part of the trisynaptic circuit, afferent inputs to the hippocampus are first sent to the dentate gyrus, which then projects to the CA3 area. The CA3 neurons then send projections to CA1. Dendrites of CA1 neurons project to the subiculum and then back to the entorhinal cortex. CA3 being an early structure in this circuit, it is the first part of the hippocampus to be affected by cholinergic overactivity. This could explain the neuronal reduction observed only in CA3 after application of low dose CPF (1/5th dermal LD_50_) for seven days. Agricultural workers chronically exposed to low-levels of CPF and other pesticides were found performing poorly on neurobehavioral tests [4]. Following occupational exposure to CPF, functional deficits in cognitive tests of abstraction, concentration and memory have also been reported [26,27]. These functional deficits can be extrapolated to be caused by prolonged exposure to low dose CPF.

Quantitative examination of the hippocampal neurons showed that consequent application of stress and CPF (1/10th and 1/5th dermal LD_50_), even for seven days, showed marked reduction in neuronal density in all areas of the hippocampus. Neuronal density in the CA3 area of the hippocampus was also shown to be significantly reduced in rats after prolonged pain stress in the form of 13 min electric shocks for 15 days [28]. It has been proposed that alterations in the cholinergic neurotransmitter systems due to stress are the initial events contributing to CNS impairment and that exacerbation of injury could occur with the concurrent exposure of stress and cholinesterase inhibitors [29]. Previous study by the authors showed that toxicity on hippocampal neurons following three-weeks-long applications of CPF at high doses (1/2 dermal LD_50_) could be exacerbated by exposure to swim stress [8]. It was reported that compared to just CPF application (1/2 dermal LD_50_), CPF with stress increased the reduction in neuronal density by 30%, 12% and 26.7% in the CA1, CA2 and CA3 areas of the hippocampus respectively. This study showed that the application of 1/10th dermal LD_50 _CPF with stress for 7 days only showed many pyknosed neurons surrounded by vacuolation of neuropil in the CA1and CA3 sub-fields of the hippocampus and the neuronal count was significantly reduced (p < 0.05) compared to the control. These changes were less apparent after application of CPF (1/10th dermal LD_50_) only. The current study has shown that stress with dermal application of CPF can cause hippocampal damage only after seven days of application at a much lower dose (1/10 dermal LD_50_). Stress has been demonstrated to increase permeability of the BBB to foreign chemicals [10]. Thus the increased permeability could have caused the increased toxicity of CPF on the hippocampal neurons observed in this study.

Following one week of CPF application at both doses (1/10th and 1/5th dermal LD_50_), GFAP expression as measured by astrocytic density was significantly increased compared to the control group. GFAP expression has been found to be increased following toxic insult to the CNS in many studies. A single subcutaneous injection (50 μg/kg bw, 1/2 LD_50_) of the cholinesterase inhibitor Sarin was found to significantly increase GFAP levels in the cerebral cortex by 269% after one hour, and to 318% after two [30]. Extended studies in rats on the effects of gestational exposure to cholinotoxicants nicotine and CPF, alone and in combination, showed increased GFAP expression in offspring in the CA1 sub-field of the hippocampus, and white matter and granular cell layer of the cerebellum [17,18]. In the present study, GFAP expression was increased in the groups receiving combined treatments of stress and CPF 0.2 dosage as compared to those just receiving CPF 0.2 dosage, but the increase was not significant. The application of swim stress with CPF 0.1 dosage did not increase the GFAP expression compared to that in CPF 0.1 dosage only. The findings suggest that toxicity resulting from stress leads to increase in GFAP expression in response to greater injury to the hippocampus with higher sub-toxic dose of CPF. Qualitative examination showed that following seven days of CPF application, GFAP expression in the astrocytes was more prominent compared to the control groups. The astrocytic processes of the groups receiving CPF were longer, and greater in number. This may be attributed to the neuroprotective effect of astrocytes limiting neuronal damage. It has been suggested that the metabolites of CPF, trichloropyridinol (TCP), exert strong toxic effects on astrocytes, compromising their neuroprotective effects and thus increasing the neurotoxicity of CPF [31]. The neuroprotective effects of astrocytes have been suggested in many studies. To assess the influence of glial cells on the neurotoxicity of OPs, aggregate brain cell cultures of foetal rat telencephalon were treated with CPF and parathion for 10 days. This *in vitro *study found that the neurotoxicity of CPF and parathion was increased in aggregate cultures deficient in glial cells [31]. When an acute dose of the OP diisopropylfluorophosphate (DFP) was injected subcutaneously into hens, the authors discovered that GFAP expression studied in total RNAs extracted from non-susceptible parts of cerebrum was upregulated from first 2 days, indicating a neuroprotective effect from anticipated imminent neurotoxicity [32].

## Conclusions

In conclusion, dermal application of low dose of CPF (1/10th dermal LD_50_) for seven days, was not capable of producing neurotoxicity in all areas of the hippocampus in the parameters of cholinesterase inhibition and neuronal density reduction. The addition of swim stress with CPF exposure caused reduction in serum cholinesterase and neuronal density of the hippocampus which was significant compared to control but not significantly different from CPF exposure alone. An interesting finding of the study was that dermal application of low dose of CPF for 7 days significantly increased GFAP expression, indicating that it can be used as a marker for CPF toxicity at the early stages. It is suggested that astrocytes may provide neuroprotective effects against CPF toxicity. Therefore, it is important that pesticide applicators should not be exposed dermally to pesticides continuously for extended periods to avoid damage to the CNS. It is also imperative that such individuals should not work under stressful conditions, as these conditions can produce neurotoxic effects.

## Competing interests

The authors declare that they have no competing interests.

## Authors' contributions

NKM and VDN designed the study. KL and AT conducted the study. NKM conducted statistical analysis of the collected data. All authors have contributed, read and approved the final manuscript.

## References

[B1] Rathinam X Kota R Thiyagar N Farmers and formulations--rural health perspective Med J Malaysia 2005 60 1 118 123 16250298

[B2] Sullivan JB Jr Blose J Sullivan JB, Krieger GR Organophosphate and carbamate insecticides Hazardous materials toxicology: Clinical principles of environmental health 1992 Williams and Wilkins, Philadelphia 1015 1026

[B3] Kiely T Donaldson D Grube A 2000 and 2001 Usage Pesticides Industry Sales and Usage, 2000 and 2001 Market Estimates. Unites States Environmental Protection Agency http://www.epa.gov/pesticides/pestsales/01pestsales/market_estimates2001.pdf Last accessed on 21 Feb 2011

[B4] Rothlein J Rohlman D Lasarev M Phillips J Muniz J McCauley L Organophosphate pesticide exposure and neurobehavioral performance in agricultural and non-agricultural Hispanic workers Environ Health Perspect 2006 114 5 691 696 10.1289/ehp.8182 16675422 PMC1459921

[B5] Kaplan JG Kessler J Rosenberg N Pack D Schaumburg HH Sensory neuropathy associated with Dursban (chlorpyrifos) exposure Neurology 1993 43 2193 2196 7694187 10.1212/wnl.43.11.2193

[B6] Abu Qare AW Abdel Rahman A Brownie C Kishk AM Abou Donia MB Inhibition of cholinesterase enzymes following a single dermal dose of chlorpyrifos and methyl parathion, alone and in combination, in pregnant rats J Toxicol Environ Health A 2001 63 3 173 189 10.1080/15287390151101529 11405414

[B7] Qiao D Seidler FJ Abreu Villaca Y Tate CA Cousins MM Slotkin TA Chlorpyrifos exposure during neurulation: cholinergic synaptic dysfunction and cellular alterations in brain regions at adolescence and adulthood Brain Res Dev Brain Res 2004 148 1 43 52 10.1016/j.devbrainres.2003.10.004 14757517

[B8] Mitra NK Nadarajah VD Siong HH Effect of concurrent application of heat, swim stress and repeated dermal application of chlorpyrifos on the hippocampal neurons in mice Folia Neuropathol 2009 47 1 60 68 19353435

[B9] Gordon CJ Leon LR Thermal stress and the physiological response to environmental toxicants Rev Environ Health 2005 20 4 235 263 16422347 10.1515/reveh.2005.20.4.235

[B10] Friedman A Kaufer D Shemer J Hendler I Soreq H Tur-Kaspa I Pyridostigmine brain penetration under stress enhances neuronal excitability and induces early immediate transcriptional response Nature Med 1996 2 1382 1385 10.1038/nm1296-1382 8946841

[B11] Norenberg M Astrocyte responses to CNS injury J Neuropathol Exp Neurol 1994 53 213 220 10.1097/00005072-199405000-00001 8176405

[B12] Abou-Donia MB Khan WA Suliman HB Abdel-Rahman AA Jensen KF Stress and combined exposure to low doses of pyridostigmine bromide, DEET, and permethrin produce neurochemical and neuropathological alterations in cerebral cortex, hippocampus, and cerebellum J Toxicol Environ Health 2004 67 2 163 192 10.1080/15287390490264802 14675905

[B13] Eng LF Ghirnikarz RS GFAP and Astrogliosis Brain Pathol 1994 4 229 237 10.1111/j.1750-3639.1994.tb00838.x 7952264

[B14] Ho G Zhang C Zhuo L Non-invasive fluorescent imaging of gliosis in transgenic mice for profiling developmental neurotoxicity Toxicol Appl Pharmacol 2007 221 1 76 85 10.1016/j.taap.2007.01.023 17350065

[B15] O'Callaghan JP Assessment of neurotoxicity: use of glial fibrillary acidic protein as a biomarker Biomed Environment Sci 1991 4 197 206 1910596

[B16] O'Callaghan JP Jensen KF Enhanced expression of glial fibrillary acidic protein and the cupric silver degeneration reaction can be used as sensitive and early indicators of neurotoxicity Neurotoxicol 1992 13 113 122 1508411

[B17] Abdel-Rahman A Dechkovskaia A Mehta-Simmons H Guan X Khan W Abou-Donia M Increased expression of glial fibrillary acidic protein in cerebellum and hippocampus: differential effects on neonatal brain regional acetylcholinesterase following maternal exposure to combined chlorpyrifos and nicotine J Toxicol Environ Health A 2003 66 21 2047 2066 10.1080/713853982 14555401

[B18] Abdel-Rahman A Dechkovskaia AM Mehta-Simmons H Sutton JM Guan X Khan WA Abou-Donia MB Maternal exposure to nicotine and chlorpyrifos, alone and in combination, leads to persistently elevated expression of glial fibrillary acidic protein in the cerebellum of the offspring in late puberty Arch Toxicol 2004 78 8 467 476 10.1007/s00204-004-0560-5 15045467

[B19] Singh A Naidu PS Gupta S Kulkarni SK Effect of Natural and Synthetic Antioxidants in a Mouse Model of Chronic Fatigue Syndrome J Med Food 2002 5 4 211 220 10.1089/109662002763003366 12639396

[B20] Abercrombie M Johnson ML Quantitative histology of Wallerian degeneration: I. Nuclear population in rabbit sciatic nerve J Anat 1946 80 37 50 PMC1272751 20996672

[B21] Jones AL Karalliedde L Boon NA, Colledge NR, Davidson SS, Walker BR Poisoning Davidson's Principles and Practice of Medicine 2006 20 Edinburgh: Churchill Livingstone 203 226

[B22] Christin D Dalon S Delamanche S Perrier P Breton P Taysse L Effects of repeated low-dose soman exposure on monoamine levels in different brain structures in mice Neurochem Res 2008 33 919 926 10.1007/s11064-007-9535-2 17994275

[B23] Prendergast MA Terry AV Jr Buccafuscoa JJ Effects of chronic, low-level organophosphate exposure on delayed recall, discrimination, and spatial learning in monkeys and rats Neurotoxicol Teratol 1998 20 2 115 122 10.1016/S0892-0362(97)00098-6 9536457

[B24] Meuling WJ Ravensberg LC Roza L van Hemmen JJ Dermal absorption of chlorpyrifos in human volunteers Int Arch Occup Environ Health 2005 78 1 44 50 10.1007/s00420-004-0558-6 15627216

[B25] Campaña AD Sanchez F Gamboa C Gómez-Villalobos Mde J De La Cruz F Zamudio S Flores G Dendritic morphology on neurons from prefrontal cortex, hippocampus, and nucleus accumbens is altered in adult male mice exposed to repeated low dose of malathion Synapse 2008 62 4 283 290 18240323 10.1002/syn.20494

[B26] Savage E Keefe T Mounce L Heaton R Lewis J Burcar P Chronic neurological sequelae of acute organophosphate pesticide poisoning Arch Environ Health 1990 43 38 45 10.1080/00039896.1988.9934372 3355242

[B27] Steenland K Jenkins B Ames R O'Malley M Chrislop D Russo J Chronic neurological sequelae to organophosphate pesticide poisoning Am J Pub Health 1995 84 731 736 10.2105/AJPH.84.5.731 PMC1615045 8179040

[B28] Shiryaeva NV Vshivtseva VV Mal'tsev NA Sukhorukov VN Vaido AI Neuron density in the hippocampus in rat strains with contrasting nervous system excitability after prolonged emotional-pain stress Neurosci Behav Physiol 2008 38 4 355 357 10.1007/s11055-008-0049-4 18401725

[B29] Pung T Klein B Blodgett D Jortner B Ehrich M Examination of concurrent exposure to repeated stress and chlorpyrifos on cholinergic, glutaminergic and monoamine neurotransmitter systems in rat forebrain region Int J Toxicol 2006 25 1 65 10.1080/10915810500527119 16510359

[B30] Damodaran TV Bilska MA Rahman AA Abou-Doni MB Sarin causes early differential alteration and persistent overexpression in mRNAs coding for glial fibrillary acidic protein (GFAP) and vimentin genes in the central nervous system of rats Neurochem Res 2002 27 5 407 415 10.1023/A:1015508132137 12064357

[B31] Zurich MG Honegger P Schilter B Costa LG Monnet-Tschudi F Involvement of glial cells in the neurotoxicity of parathion and chlorpyrifos Toxicol Appl Pharmacol 2004 201 2 97 104 10.1016/j.taap.2004.05.003 15541749

[B32] Damodaran TV Abou-Donia MB Alterations in levels of mRNAs coding for glial fibrillary acidic protein (GFAP) and vimentin genes in the central nervous system of hens treated with diisopropyl phosphorofluoridate (DFP) Neurochem Res 2000 25 6 809 816 10.1023/A:1007565407341 10943999

